# A 14-Year Retrospective Analysis of Endogenous Endophthalmitis in a Tertiary Referral Center of Southern Thailand

**DOI:** 10.1155/2020/6689081

**Published:** 2020-12-15

**Authors:** Patama Bhurayanontachai, Phingphan Klongthanakit

**Affiliations:** Department of Ophthalmology, Faculty of Medicine, Prince of Songkla University, 15 Karnjanawanich Road, Hat Yai, Songkhla 90110, Thailand

## Abstract

**Purpose:**

To investigate patient characteristics, clinical features, common causative organisms, and visual acuity outcomes in endogenous endophthalmitis.

**Methods:**

This was a retrospective chart analysis of patients with endogenous endophthalmitis between January 2006 and December 2019. Collected data included basic patient characteristics, presenting symptoms, causative organisms, treatments, and 3-month and 1-year visual outcomes.

**Results:**

Twenty-nine eyes of 27 patients were included in the study. The mean age of the patients was 45.4 ± 19.9 years, and 63% were female. Visual acuity at presentation ranged from counting fingers to no light perception. Systemic comorbidities presented in 66.7% of the patients, the majority of which were related to diabetes mellitus (48.1%). The most common primary infection was a urinary tract infection. Positive blood cultures were identified in 48.1% of patients, and positive cultures from vitreous and aqueous samples were identified in 59.3% and 31.6% of eyes, respectively. Among all the specimens, Gram-positive bacteria were identified in 55.5%, Gram-negative bacteria in 22.2%, fungi in 14.8%, and mixed organisms in 7.4%. Among ocular specimens, 61.1% contained Gram-positive organisms, 16.7% contained Gram-negative organisms, and 22.2% contained fungi. *Streptococcus spp.* was the most common causative organism. From 29 eyes, 18 (62.1%) underwent vitrectomy, and 12 (42.9%) underwent either evisceration or enucleation. Positive vitreous culture was significantly associated with unfavorable final visual outcome. Final visual acuity ranged from 20/125 to no light perception. Although visual improvement at 3 months was significantly better in younger patients, this had no impact on final visual outcome at 1 year.

**Conclusion:**

Eyes with positive vitreous cultures had significantly poorer visual outcomes. Despite full treatment coverage, visual prognosis was extremely poor and the rates of blindness and evisceration/enucleation were still high.

## 1. Introduction

Endogenous endophthalmitis is defined as an eye infection involving the vitreous and/or aqueous humors, arising from bacteremic or fungemic seeding from other organs. It is a rare, but sight-threatening, condition characterized by severe inflammation of the intraocular spaces. This condition is usually caused by hematogenous spread of organisms from a distant source through the blood-ocular barrier; it is associated with underlying factors such as diabetes mellitus, indwelling catheters, malnourishment, immunocompromised hosts, and intravenous drug abuse [1–4]. Clinical course of the condition is typically acute with rapid progression, requiring prompt diagnosis and management to preserve acceptable vision.

In East Asian hospitals, Gram-negative bacteria are the main causative organism in endogenous endophthalmitis, responsible for 54.4%–78% cases, with *Klebsiella pneumoniae* being the most common species [3, 5, 6]. Conversely, in European and North American countries, Gram-positive bacteria are more commonly involved in the condition than Gram-negative bacteria [3, 7]. A positive blood culture was reported in 37.1%–94% of patients with endogenous endophthalmitis, whereas a positive intraocular culture was reported in 22.3%–58% [1–3, 6, 7]. However, in a series of endophthalmitis cases in children, the number of positive blood cultures was as low as zero [4].

The mainstay of treatment for most patients usually involves the administration of both intraocular and systemic antibiotics [1, 2, 4]. Favorable outcomes can be achieved by early intravitreal and systemic antibiotics only without routine vitrectomy [8]. Nevertheless, pars plana vitrectomy plays a role in severe or worsening cases.

Due to the rareness and complexity of the condition as well as the guarded visual prognosis, more information is required on this condition. Therefore, this study aimed primarily to demonstrate characteristics and results of endogenous endophthalmitis at 3 months and 1 year after treatment. The secondary aim was to evaluate which factors may affect visual outcomes.

## 2. Patients and Methods

### 2.1. Study Design

This retrospective study was carried out in accordance with the Declaration of Helsinki and was approved by the Ethics Committee of the Faculty of Medicine, Prince of Songkla University, Songkhla, Thailand (REC number 58-101-02-4). Informed consent was waived due to the retrospective design of the study.

### 2.2. Participants

Songklanagarind Hospital is a tertiary teaching hospital in southern Thailand. Patients with any types of endophthalmitis between January 2006 and December 2019 were identified by reviewing the hospital's electronic medical record database. Endogenous endophthalmitis was defined as the presence of intraocular inflammation with no history of previous trauma or surgery. Patients were included if they had either a positive intraocular culture (aqueous or vitreous) or a positive systemic culture (blood, body fluid, or pus). Patients were excluded if they had uveitis or a potential exogenous cause of infection, such as recent ocular trauma or intraocular surgery, within 1 year prior to presentation.

### 2.3. Data Collection

The collected data consisted of general patient characteristics, baseline visual acuity (VA), microbiologic reports of causative organisms, treatments, and final visual outcomes. Best-corrected visual acuity (BCVA), measured using the Early Treatment Diabetic Retinopathy Study chart, was converted into a logarithm of the minimum angle of resolution (logMAR) VA for numeric comparison and statistical analysis. Ultra-low VA such as counting fingers, hand motion, light projection/light perception, and no light perception were assigned VA values of 20/4000 (2.3 logMAR), 20/8000 (2.6 logMAR), 20/16000 (2.9 logMAR), and 20/32000 (3.2 logMAR), respectively, in keeping with previous literature [9]. Early (3 months) and late (1 year) visual outcomes were classified into two groups: one group with improvement of vision and another without. If the BCVA after treatment was better than that of the baseline, the visual outcome had an “improvement.” If the BCVA after treatment was the same or worse than that of the baseline, the visual outcome had “no improvement.”

### 2.4. Management of Endogenous Endophthalmitis

Every intraocular sample was taken for organism identification under sterile technique. Topical application of 5% povidone iodine solution was used to paint and rinse around the eye before the surgery. Aqueous tapping was performed at the peripheral cornea using 30-gauged needle on a tuberculin syringe. Vitreous tapping was performed at the pars plana area, 3.5–4 mm posterior to the corneal limbus, using either 23- or 25-guaged needle on a 3-ml syringe or a standard 23-guaged vitrectomy cutter (Ultravit^®^ vitrectomy probe, Alcon laboratories, Inc., Forth Worth, TX). Approximately 0.2–0.3 ml of specimen was obtained.

All cases were initially treated with systemic antimicrobial agents. Empirical intravitreal antibiotics, consisting of 1 mg vancomycin and 2.25 mg ceftazidime every 2-3 days, were administered to patients too ill to undergo an immediate standard vitrectomy. Subsequent intravitreal antibiotic injections, including during postvitrectomy stage, were decided upon based on the results of antibiotic susceptibility testing.

### 2.5. Statistical Analysis

The Kolmogorov–Smirnov test was used to test that the data were normally distributed. Continuous variables were reported as mean ± standard deviation (SD) or median and interquartile range (IQR) depending on the distribution of data. Categorical variables were reported as number and percentage.

Paired *t*-test was used to compare the BCVA before and after treatment. Independent *t*-test, Mann–Whitney test, Chi-squared test, and Fisher's exact test were appropriately chosen to compare the clinical characteristics between the two visual outcome groups. In each case, comparisons were considered statistically significant if the *p* value was <0.05. All data were analyzed using MedCalc Statistical Software version 19.0.5 (MedCalc Software bvba, Ostend, Belgium).

## 3. Results

Of 802 patients diagnosed with endophthalmitis during the study period, 27 (3.4%) were included in this study. The mean age of the patients was 45.4 ± 19.9 years, and 63% were female. Uniocular involvement was found in 92.6% of cases and was more predominant in left eyes. Bilateral endogenous endophthalmitis was observed in 2 of the 27 cases (7.4%). Symptoms at presentation included decreased vision (100%), red eye (96.4%), ocular pain (74.1%), fever (37%), and headache or body pain (22.2%). Ophthalmic signs included hypopyon or presence of anterior chamber cells (31%), lid swelling or proptosis (20.7%), and limited ocular movement (20.7%). Two patients (7.4%) initially presented with panophthalmitis.

The median duration of symptoms prior to admission was 8 (3–18.5) days, with the exception of one patient in whom ophthalmic symptoms were detected by the caring doctors 16 days after admission and intubation for systemic treatment. The median interval before referral was 3 (0–11) days. The median duration of symptoms prior to admission was the shortest in patients infected with Gram-positive and Gram-negative bacteria, at 5 (2–10.3) and 8 (5.8–18.5) days, respectively. Patients infected with fungus generally presented late, with a median duration of symptoms of 24 (8–61) days.

All patients presented with severe visual deterioration, ranging from counting fingers to no light perception, with a median BCVA at baseline of 2.9 (2.6–3.2) logMAR. Initial VA could not be obtained in two cases, one in a patient with Down syndrome with delayed development, and the other in a patient who was intubated. A summary of baseline characteristics of patients is presented in Table 1. One patient underwent an enucleation and died due to multiple organ failure during in-hospital treatment. One patient was lost to follow-up after discharge and two further patients were lost to follow-up after 3 months.

Growth of organisms in blood culture was observed in 13 of 27 (48.1%) patients. Regardless of blood culture results, the primary site of infection could be identified in 11 patients (40.7%). A summary of primary sites and causative organisms is shown in Table 2. Overall, causative organisms comprised Gram-positive bacteria in 15 patients (55.5%), Gram-negative bacteria in 6 patients (22.2%), fungus in 4 patients (14.8%), and mixed organisms in 2 patients (7.4%). Organisms were detected in 16 (59.3%) of 27 vitreous samples and in 6 (31.6%) of 19 aqueous samples. Identifiable organisms from ocular specimens were 80% consistent with those from the primary sites. From 18 culture-proven ocular specimens, 11 (61.1%) contained Gram-positive organisms, 3 (16.7%) contained Gram-negative organisms, and 4 (22.2%) contained fungi.

Patients received treatment at 1.7 days (mean) after admission or consultation. Twelve patients (13 eyes) received intravitreal antibiotics at least once followed by a vitrectomy or enucleation/evisceration. Four patients (4 eyes) whose conditions were not suitable for surgery received only intravitreal antibiotics. Two patients (2 eyes) were treated with early enucleation due to an initial diagnosis of panophthalmitis. Primary vitrectomy without preceding intravitreal antibiotics was performed in nine eyes of nine patients; however, 3 of these required subsequent evisceration (1 eye) or enucleation (2 eyes). One eye required a repeated vitrectomy after one week due to recurrent vitreous haze. From 6 eyes that required a subsequent evisceration or enucleation after the vitrectomy, 2 had intraocular samples showing positive cultures for Gram-positive cocci (*Beta-Streptococcus* and *Staphylococcus* spp.), 2 for Gram-negative bacteria (*K. pneumoniae* and *P. aeruginosa*), and one for fungus (*Curvularia* spp.); the intraocular sample for the remaining eye had a negative culture. The indications for evisceration or enucleation were panophthalmitis and painful blinded eye.

Median BCVA values at 3 months and 1 year post-treatment were 3.2 (2.6–3.2) logMAR and 3.2 (2.9–3.2) logMAR, respectively. No significant differences were observed in the change of BCVA from baseline, either at 3 months (*p*=0.79) or at 1 year (*p*=0.68). Counting until each patient's last visit, 18 eyes (62.1%) ultimately had no light perception. Final visual improvement was reported in only 4 eyes (13.8%). After excluding the 12 eviscerated/enucleated eyes and the 4 eyes lost to follow-up, the median BCVA was 2.9 (2.4–3.2) logMAR at 1 year after treatment. No significant difference between final BCVA and baseline was observed, either before or after excluding eviscerated/enucleated eyes (*p*=0.68 and *p*=0.44, respectively).

Late complications included cataract progression, retinal detachment, corneal decompensation, and phthisis bulbus.

Correlations between patient characteristics and visual outcomes are shown in Table 3. At 3 months, VA improvement was significantly found in younger patients at a mean age of 24.4 years, compared with 49.8 years in the no improvement group (*p*=0.01). However, there was no correlation between mean age and VA improvement at 1 year (*p*=0.46). A positive vitreous culture was a strong factor, significantly associated with poor late visual outcome (*p*=0.01), although this association tended toward statistical significance by 3 months postoperatively (*p*=0.05). Underlying disease and source of infection were not related to final visual outcome.

## 4. Discussion

In our study, endogenous endophthalmitis contributed to 3.4% of all cases of endophthalmitis, a finding that was similar to that in other reports, varying from 2% to 18.5% [1, 5, 10–12]. Most of the patients (66.7%) had one or more predisposing factors such as diabetes mellitus, hypertension, and kidney-ureter-bladder system disorders. The primary source of septicemia could only be identified in 40.7% of patients in this study. The source of infection could be identified in 38%–75% patients in other studies [6, 12, 13]. Our study found that urinary tract infection was the most common primary site of infection, followed by abscess in the musculoskeletal system. None of the patients had a hepatobiliary tract disorder.

In our study, a positive culture result was observed in 59.3% of vitreous samples and in 31.6% of aqueous samples. These results from vitreous culture were slightly higher than in previous literature [1, 3, 6, 7]. Furthermore, results from aqueous culture were much higher than a previous case series [3]. The causative organisms of endogenous endophthalmitis reported in Asian populations, including central Thailand, are predominantly Gram-negative organisms, with the most common being *Klebsiella pneumoniae* [3, 5, 6, 11]. In the literature, liver abscesses accounted for most of the metastatic origins of *Klebsiella pneumoniae*. In contrast, our study showed that gram-positive bacteria were the most common causative organisms. Differences in endemic variability may be the reason behind our findings. A report from northeastern Thailand also demonstrated a higher prevalence of Gram-positive organisms [10]. Reports from China have shown that causative organisms vary between regions [5, 14, 15].

The current standard treatment for endogenous endophthalmitis consists of intravenous antibiotics to target the systemic source of infection, alongside intravitreal antibiotics as the local control. All patients in the study received both intravenous and intravitreal antibiotics following their microbial sensitivity tests. The mean interval before commencing ophthalmic treatment was as early as 1.7 days after admission or consultation but the median duration of symptoms prior to the admission was 8 (3–18.5) days and the median interval before referral was 3 (0–11) days. This may be the reason why 72% of eyes ultimately had a VA of no light perception and 84% of eyes could not maintain their ambulatory vision. Therefore, we suggest an early diagnosis and referral for patients with suspected endogenous endophthalmitis. Vitrectomy can mechanically remove infecting bacteria, toxins, cytokines, inflammatory cells, and membranes. Excellent visual outcomes following vitrectomy have previously been reported, especially in cases of fungal endogenous endophthalmitis [4, 13, 14, 16]. However, many critically ill patients in this study were contraindicated for surgery due to multiple comorbidities, and 41.4% of eyes received at least one injection prior to the vitrectomy or evisceration/enucleation. In total, 13.8% of eyes received only intravitreal injections without vitrectomy.

Patients with a longer duration of symptoms prior to admission tended to have better visual improvement than those with a shorter duration of symptoms, although this difference was not statistically significant. This could be a result of a more indolent nature of the infecting organism leading to a slower rate of retinal damage and providing time for adequate intervention. Muda et al. [6] previously found that a good initial BCVA was an independent factor for a good final outcome. All patients in our study presented with an initial VA of less than able to read and final visual prognosis was unsatisfied. More than 60% of eyes ultimately had no light perception.

Our study found that a positive vitreous culture was also correlated with poor visual outcome, particularly in the late postoperative period. A positive culture may reflect the large load of organism inside the eye and the severity of intraocular damage from the infecting organism.

There have been a few case reports regarding endogenous endophthalmitis in young healthy patients, [17–19] and after common dental or gynecological procedures and postpartum [20–22]. Our study surprisingly found that one-third of cases were both young and healthy patients. They could be infected by any type of organisms. One previously healthy patient had surgical treatment for hemorrhoids prior to the onset of an *Aeromonas* septicemia, pyomyositis, and finally endophthalmitis. Caution should be exercised about the fact that the common invasive procedures can result in transient bacteremia which may lead to serious consequences.

Long-term complications included cataract progression, retinal detachment, and phthisis bulbus, in keeping with results from previous studies [3, 13]. These complications compromised final visual outcome although globe salvage had been achieved.

Our study contained some limitations that may compromise the clinical implications of its findings. Firstly, this observational retrospective study contained missing data and the efficacy of treatment modalities could not be applied. Secondly, cases referred to this tertiary hospital were usually severe and complicated, which may have caused selection bias resulting in grave visual outcomes.

## 5. Conclusion

Endogenous endophthalmitis accounted for 3.3% of all endophthalmitis cases diagnosed in our study. *Streptococcus* spp. was the most common causative organism and septicemia was the most common source of infection. Endogenous endophthalmitis is generally associated with poor VA outcomes. Younger age was a favorable factor for early visual improvement after treatment, while a positive vitreous culture was correlated with a poorer long-term visual prognosis. Even though early treatment was administered with intravitreal antibiotics and vitrectomy, the rate of enucleation and evisceration remained high.

## Figures and Tables

**Table 1:** Baseline characteristics of the patients with endogenous endophthalmitis.

Parameters	Number of patients (*n* = 27)

Sex:	
Male	10 (37%)
Female	17 (63%)
Presence of underlying disease:	18 (66.7%)
Diabetes mellitus	13 (48.1%)
Hypertension	8 (29.6%)
KUB system	7 (25.9%)
Heart disease	3 (11.1%)
Dyslipidemia	2 (7.4%)
Liver disease	2 (7.4%)
HBV infection	2 (7.4%)
Lung disease	1 (3.7%)
Graves' disease	1 (3.7%)
Hematologic disorder	1 (3.7%)
HIV infection	1 (3.7%)
Carcinoma	1 (3.7%)
Presence of primary source of infection:	11 (40.7%)
UTI	5 (18.5%)
Abscess at any body part	4 (14.8%)
Lung	1 (3.7%)

	Number of eyes (*n* = 29)
Laterality:	
Right	12 (41.4%)
Left	17 (58.6%)
Presenting VA:	
No light perception	10 (34.5%)
Light perception	5 (17.2%)
Projection of light	2 (6.9%)
Hand movement	8 (27.6%)
Counting finger	2 (6.9%)
Cannot be evaluated	2 (6.9%)
Positive ocular culture:	25 (86.2%)
Gram positive	16 (55.2%)
Gram negative	7 (24.1%)
Fungus	6 (20.7%)
Treatment:	
Intravitreal antibiotics prior to PPV or evisceration/enucleation	13 (44.8%)
PPV	18 (62.1%)
Evisceration/enucleation	12 (41.4%)

A/C: anterior chamber; EOM: extraocular muscle movement; IOP: intraocular pressure; KUB: kidney ureter and bladder; PPV: pars plana vitrectomy; SD: standard deviation; UTI: urinary tract infection; VA: visual acuity.

**Table 2:** Clinical summary of patients with endogenous endophthalmitis.

Case	Sex/age/eye	Underlying condition	Systemic diagnosis	Organism	Culture				Initial VA	VA at 3 months	VA at 1 year
					Vitreous	Aqueous	Blood	Others			
1	M/69/R	DM, HT, ESRD	—	*S. aureus*	+	NA	—	NA	No PL	No PL	No PL
2	M/69/L	DM, HT, DLP	Psoas abscess	*Beta-Streptococcus gr. B*	+	NA	—	+ (pus)	No PL	No PL	No PL
3	F/50/R	DM, ESRD, dilated cardiomyopathy with severe TR, anemia	Septicemia, UTI, infected CAPD	*K. pneumoniae ESBL*, *P. aeruginosa*,* S. aureus (conjunctiva)*	NA	—	—	+ (peritoneal fluid, urine, subconjunctival pus)	No PL	Dead	Dead
4	F/32/L	None	—	*P. aeruginosa*,* Xanthomonas*	+	NA	—	NA	PJ	No PL	No PL
5	F/75/L	None	Septicemia	*B-Streptococcus spp. (not gr A*, *B*, *D)*, *Staphylococcus spp. (urine)*	+	NA	+	+ (urine)	No PL	No PL	No PL
6	F/25/L	DM, AKI, chronic osteomyelitis	UTI, pyelonephritis	*K. pneumoniae ESBL*	+	NA	—	+ (urine)	No PL	No PL	No PL
7	F/33/L	DM, HBV, Down syndrome	Septicemia	*S. epidermidis*	—	—	+	—	NA	HM	PL
8	F/8/R	None	—	*Paecilomyces spp.*	—	+	—	—	PL	HM	No PL
9	M/42/L	None	—	*P. aeruginosa*	+	—	—	—	No PL	No PL	No PL
10	M/35/L	None	—	*Phaeoacremonium spp.*	+	+	—	NA	HM	HM	PL
11	M/43/R	Cirrhosis, IgA nephropathy, HBV	Septicemia	*P. aeruginosa*	—	NA	+	NA	No PL	No PL	NA
12	M/66/R	DM, HT	Septicemia	*Beta-Streptococcus gr. B*	+	+	+	NA	HM	No PL	No PL
13	F/41/L	DM	—	*Beta-Streptococcus gr. B*	+	NA	—	—	PL	No PL	No PL
14	F/42/R	DM, HT, hyperthyroidism	UTI, pneumonia	*K. pneumoniae (urine)*, *candida (sputum)*, *Carvularia spp. (vitreous)*	+	NA	—	+ (urine, sputum)	PL	No PL	No PL
15	F/31/L	HIV	Septicemia	*P. marneffei*	—	—	+	—	FC1F	20/50	5/200
16	F/52/L	DM	Bacteremia, septic arthritis both elbows	*Beta-Streptococcus gr. B*, *S. Epidermidis*	+	+	+	NA	PJ	No PL	No PL
17	F/76/L	HT, cirrhosis	Septicemia	*Beta-Streptococcus gr. B*, *Candida spp. (urine)*	+	—	+	+ (urine)	No PL	No PL	No PL
18	F/58/L	Cervical spondylitis	Septicemia, UTI, epidural abscess at C-spine	*Beta-Streptococcus gr. B*, *Staphylococcus spp. (urine)*	+	+	+	+ (urine)	HM	No PL	No PL
19	M/12/L	None (history of pleural effusion)	—	*Corynebacterium spp.*	+	—	—	—	HM	20/40	HM
20	M/61/L	Ebstein anomaly with persistent foramen ovale	—	*Staphylococcus coag-negative*	—	+	—	NA	PL	No PL	No PL
21	M/51/R	None	Septicemia, brain abscess	*S. saprophyticus*, *E. coli (sputum)*	—	—	+	+ (sputum)	No PL	NA	NA
	M/51/L				NA	NA			HM	NA	NA
22	F/44/R	DM, HT, renal stone	UTI	*C. albicans*	—	—	—	+ (urine)	FC1F	HM	NA
	F/44/L				+	—			HM	PJ	NA
23	F/9/L	None	—	*S. pneumonia*	+	NA	—	—	PL	PJ	PJ
24	F/72/R	DM, AKI, CA cervix, (history of *E. coli* septicemia)	Septicemia, renal abscess	*K. pneumoniae*	—	—	+	+ (urine)	NA	No PL	No PL
25	F/62/R	DM, HT, DLP	Septicemia, pyomyositis	*Beta-Streptococcus gr. B*	—	—	+	+ (pus)	HM	20/200	5/200
26	F/23/R	None (after hemorrhoid treatment)	Septicemia, pyomyositis	*A. hydrophila*	—	—	+	+ (pus)	No PL	No PL	No PL
27	M/46/R	DM, HT, ESRD	Septicemia, UTI	*S. aureus*	—	—	+	—	HM	HM	20/125

AKI: acute kidney injury, CAPD: continuous ambulatory peritoneal dialysis, DLP: dyslipidemia, DM: diabetes mellitus, ESRD: end-stage renal disease, F: female, FC1F: finger counting at 1-foot distance, HBV: hepatitis B virus, HIV: human immunodeficiency virus, HM: hand motion, HT: hypertension, L: left eye, M: male, NA: no data, PJ: projection of light, PL: perception of light, R: right eye, TB: tuberculosis, TR: tricuspid regurgitation, UTI: urinary tract infection, and VA: visual acuity.

**Table 3:** Correlation between patient characteristics and visual outcomes.

	VA Status at 3 months		*p* value	VA Status at 1 year		*p* value
	Improvement (*n* = 5)	No improvement (patient, *n* = 18) (eye, *n* = 19)		Improvement (*n* = 4)	No improvement (*n* = 17)	
Mean age (SD)	24.4 (23.0)	49.8 (16.7)	0.01^†^	37.0 (22.6)	46.1 (21.7)	0.46^†^
Median time to admission, d (IQR)	17 (7.8–40)	6 (2.8–18.8)	0.16^‡^	20 (6.5–32)	5 (2–17.8)	0.28^‡^
Female, *n* (%)	4 (80)	10 (55.6)	0.33^*∗*^	3 (75)	10 (58.8)	0.56^*∗*^
Left eye, *n* (%)	3 (60)	12 (63.2)	0.89^*∗*^	2 (50)	12 (70.6)	0.44^*∗*^
Proptosis and lid swelling	1 (20)	4 (21.1)	0.96^*∗*^	0 (0)	5 (29.4)	0.22^*∗*^
EOM limitation	0 (0)	4 (21.1)	0.27^*∗*^	0 (0)	4 (23.5)	0.29^*∗*^
Presence of any underlying disease	3 (60)	12 (66.7)	0.79^*∗*^	2 (50)	11 (64.7)	0.59^*∗*^
DM	1 (20)	9 (50)	0.24^*∗*^	2 (50)	7 (41.2)	0.75^*∗*^
Hypertension	1 (20)	7 (38.9)	0.44^*∗*^	2 (50)	5 (29.4)	0.44^*∗*^
KUB system	0 (0)	5 (27.8)	0.85^*∗*^	1 (25)	2 (11.8)	0.46^*∗*^
Presence of primary source	2 (40)	12 (66.7)	0.29^*∗*^	3 (75)	9 (52.9)	0.43^*∗*^
Septicemia	1 (20)	7 (38.9)	0.44^*∗*^	2 (50)	5 (29.4)	0.44^*∗*^
UTI	0 (0)	4 (22.2)	0.78^*∗*^	1 (25)	2 (11.8)	0.34^*∗*^
Positive hemoculture	2 (40)	8 (44.4)	0.86^*∗*^	3 (75)	6 (35.3)	0.16^*∗*^
Positive vitreous culture	2 (40)	16 (84.2)	0.05^*∗*^	1 (25)	15 (88.2)	0.01^*∗*^
Positive aqueous culture	1 (25) (*n* = 4)	6 (54.5) (*n* = 11)	0.32^*∗*^	0 (0) (*n* = 3)	6 (60) (*n* = 10)	0.08^*∗*^
Presence of Gram-positive bacteria	3 (60)	10 (52.6)	0.77^*∗*^	3 (75)	10 (58.8)	0.56^*∗*^
Presence of Gram-negative bacteria	0 (0)	6 (31.6)	0.16^*∗*^	0 (0)	5 (29.4)	0.23^*∗*^
Presence of fungus	2 (40)	4 (21.1)	0.39^*∗*^	1 (25)	3 (17.6)	0.74^*∗*^

^†^Independent *t*-test, ^‡^Mann–Whitney test, ^*∗*^Fisher's exact test, DM: diabetes mellitus, EOM: extraocular muscle movement, KUB system: kidney-ureter-bladder system, and UTI: urinary tract infection.

## Data Availability

The datasets used and analyzed in the current study are available from the corresponding author on reasonable request.

## References

[1] Jackson T. L., Eykyn S. J., Graham E. M., Stanford M. R. (2003). Endogenous bacterial endophthalmitis: a 17-year prospective series and review of 267 reported cases. *Survey of Ophthalmology*.

[2] Jackson T. L., Paraskevopoulos T., Georgalas I. (2014). Systematic review of 342 cases of endogenous bacterial endophthalmitis. *Survey of Ophthalmology*.

[3] Cho H., Shin Y. U., Siegel N. H. (2018). Endogenous endophthalmitis in the American and Korean population: an 8-year retrospective study. *Ocular Immunology and Inflammation*.

[4] Maitray A., Rishi E., Rishi P. (2019). Endogenous endophthalmitis in children and adolescents: case series and literature review. *Indian Journal of Ophthalmology*.

[5] Duan F., Wu K., Liao J. (2016). Causative microorganisms of infectious endophthalmitis: a 5-year retrospective study. *Journal of Ophthalmology*.

[6] Muda R., Vayavari V., Subbiah D., Ishak H., Adnan A., Mohamed S. O. (2018). Endogenous endophthalmitis: a 9-year retrospective study at a tertiary referral hospital in Malaysia. *Journal of Ophthalmic Inflammation and Infection*.

[7] Bjerrum S. S., la Cour M. (2017). 59 eyes with endogenous endophthalmitis- causes, outcomes and mortality in a Danish population between 2000 and 2016. *Graefe’s Archive for Clinical and Experimental Ophthalmology*.

[8] Yonekawa Y., Chan R. P., Reddy A. K., Pieroni C. G., Lee T. C., Lee S. (2011). Early intravitreal treatment of endogenous bacterial endophthalmitis. *Clinical & Experimental Ophthalmology*.

[9] Scott I. U., Schein O. D., West S., Bandeen-Roche K., Enger C., Folstein M. F. (1994). Functional status and quality of life measurement among ophthalmic patients. *Archives of Ophthalmology*.

[10] Yospaiboon Y., Intarapanich A., Laovirojjanakul W. (2018). Factors affecting visual outcomes after treatment of infectious endophthalmitis in northeastern Thailand. *Clinical Ophthalmology*.

[11] Silpa-Archa S., Ponwong A., Preble J. M., Foster C. S. (2018). Culture-positive endogenous endophthalmitis: an eleven-year retrospective study in the central region of Thailand. *Ocular Immunology and Inflammation*.

[12] Chung K. S., Kim Y. K., Song Y. G. (2011). Clinical review of endogenous endophthalmitis in Korea: a 14-year review of culture positive cases of two large hospitals. *Yonsei Medical Journal*.

[13] Modjtahedi B. S., Finn A. P., Barb S. M. (2019). Characteristics and outcomes of endogenous endophthalmitis. *Ophthalmology Retina*.

[14] Zhang Y.-Q., Wang W.-J. (2005). Treatment outcomes after pars plana vitrectomy for endogenous endophthalmitis. *Retina*.

[15] Duan F., Yang Y., Yuan Z., Zheng Y., Cheng Z., Lin X. (2017). Clinical features and visual acuity outcomes in culture-positive endogenous fungal endophthalmitis in southern China. *Journal of Ophthalmology*.

[16] Behera U. C., Budhwani M., Das T. (2018). Role of early vitrectomy in the treatment of fungal endophthalmitis. *Retina*.

[17] Shankar K., Gyanendra L., Hari S., Dev Narayan S. (2009). Culture proven endogenous bacterial endophthalmitis in apparently healthy individuals. *Ocular Immunology and Inflammation*.

[18] Duong T., Shahbazi S., Lee S. (2018). Endogenous group A streptococcal endophthalmitis in a healthy 42-year-old man: a case report. *The Permanente Journal*.

[19] Kostick D. A., Foster R. E., Lowder C. Y., Meyers S. M., McHenry M. C. (1992). Endogenous endophthalmitis caused by Candida albicans in a healthy woman. *American Journal of Ophthalmology*.

[20] Mali J. O., Falk N. S., Mali Y. P., Mencias L. (2015). Endogenous endophthalmitis with iris abscess after routine dental cleaning. *JAMA Ophthalmology*.

[21] Chang T. S., Chen W. C., Chen H. S., Lee H. W. (2002). Endogenous Candida endophthalmitis after two consecutive procedures of suction dilatation and curettage. *Chang Gung Medical Journal*.

[22] Wu J.-S., Chen S.-N., Hwang J.-F., Lin C.-J. (2011). Endogenous mycotic endophthalmitis in an immunocompetent postpartum patient. *Retinal Cases & Brief Reports*.

